# Changes in body perception following virtual object manipulation are accompanied by changes of the internal reference scale

**DOI:** 10.1038/s41598-023-34311-8

**Published:** 2023-05-02

**Authors:** Wladimir Kirsch, Wilfried Kunde

**Affiliations:** Department of Psychology, University of Würzburg, Röntgenring 11, 97070 Würzburg, Germany

**Keywords:** Human behaviour, Perception

## Abstract

Changes in body perception often arise when observers are confronted with related yet discrepant multisensory signals. Some of these effects are interpreted as outcomes of sensory integration of various signals, whereas related biases are ascribed to learning-dependent recalibration of coding individual signals. The present study explored whether the same sensorimotor experience entails changes in body perception that are indicative of multisensory integration and those that indicate recalibration. Participants enclosed visual objects by a pair of visual cursors controlled by finger movements. Then either they judged their perceived finger posture (indicating multisensory integration) or they produced a certain finger posture (indicating recalibration). An experimental variation of the size of the visual object resulted in systematic and opposite biases of the perceived and produced finger distances. This pattern of results is consistent with the assumption that multisensory integration and recalibration had a common origin in the task we used.

## Introduction

It has been previously demonstrated that the perception of the human body can be distorted under certain conditions as indicated, e.g., by several bodily illusions^1^. For example, a covered hand feels to be closer to a fake hand when movements of the fake hand are controlled by movements of the real hand^2^. This and similar phenomena likely arise because observers tend to integrate related yet spatially discrepant multisensory signals, such as proprioceptive signals from the real hand and visual signals from the fake hand^3^.

Consider the example of grasping an object, where observers receive proprioceptive feedback of the size of a touched object that is somewhat discrepant to the visual object size. When the size of a grasped visual object and the magnitude of observer’s hand opening do not correspond, the perception of the hand opening and of the object size change in a systematic and predictable manner^4,5^. In particular, the current visual object size attracts the felt hand opening and vice versa, the currently felt hand opening attracts the visual object size. Such biases usually vary depending on how precise the respective signals are (“reliability weighting”) and how strong their perceived relation is (“unity assumption”)^6–9^. These regularities hold for diverse interactions with the environment including virtual object manipulations^10–12^. For example, in a cursor control task, participants control a cursor displayed in the fronto-parallel plane by arm movements performed on a horizontal plane^13,14^. When the direction of the cursor movement is rotated relative to the direction of the hand movement, the felt hand direction is biased towards the currently seen cursor direction and, vice versa, the seen cursor direction is biased towards the currently felt hand direction (though to a lesser extent).

Recent research on visuo-motor learning (or adaptation) has revealed similar observations which, however, are construed in a different manner, namely as changes of sensory reference frames, which is often labelled as (sensory, motor or sensorimotor) “recalibration”, “remapping” or “realignment”. In essence, the internal reference scales underlying perception (or motor control) are assumed to be altered in a certain way following an experience of a discrepancy between related multisensory stimuli. Consider for example that after learning to reach with misaligned visual feedback of the hand, the felt hand location is attracted by the location of visual feedback of the hand^15–17^ and vice versa, the perception of the visual feedback location is attracted by the felt hand position^18,19^. These effects are very similar to those mentioned in the previous paragraph on multisensory integration. The main difference is that recalibration is supposed to be a matter of learning, thus to arise only after repeated or prolonged experience with conflicting spatial information, whereas multisensory integration is supposed to happen even with single encounters of discrepant sensory input. However, more recently, it has been demonstrated that changes in hand perception occur very early during visuomotor learning and can be observed even after a single exposure to multisensory discrepancies^20–22^. These results seem to correspond well with findings from the audiovisual domain demonstrating that recalibration can also occur following a single exposure to multisensory discrepancy^23–27^.

This apparent similarity between effects observed in multisensory setups on the one hand and learning setups on the other hand is intriguing because it raises the possibility that what is called recalibration could be a part of what is called multisensory integration and, vice versa, recalibration could obey to the same basic principles as multisensory integration (see above). In other words, the observed biases in perception and learning might reflect the same phenomenon measured with different procedures, at least to some extent. This assumption is not new and already received some support. In particular, Smeets and colleagues^28^ argued that some findings in visuo-motor learning studies usually ascribed to recalibration of the senses could be explained by a change of the relative weights assigned to the senses during multisensory integration. The authors showed that such an optimal integration model accounts for a drift in movement endpoints after removing of visual feedback without any assumptions regarding recalibration, but see^29^. In a similar vein, it has been demonstrated that the movements of the thumb and index finger of the same hand can adapt to opposite prism displacements and multisensory integration was assumed to be a likely source of this adaptation^30^. Moreover, this adaptation in pointing of single digits proved to transfer to grasping with these digits^31^ but not to manual estimations^32^ indicating no realignment or recalibration of vision and/or proprioception in this type of prism adaptation, see also^33^.

The present study aimed to contribute to this still unresolved issue of whether and how indicators of multisensory integration are related to the indicators of recalibration. The primary question of interest was whether the same sensorimotor experience prompts both, the type of changes in body perception that are indicative of multisensory integration as well as changes in body perception that are indicative of a distortion in the internal reference scale (i.e. of recalibration).

## Experiment 1

**Figure 1:**
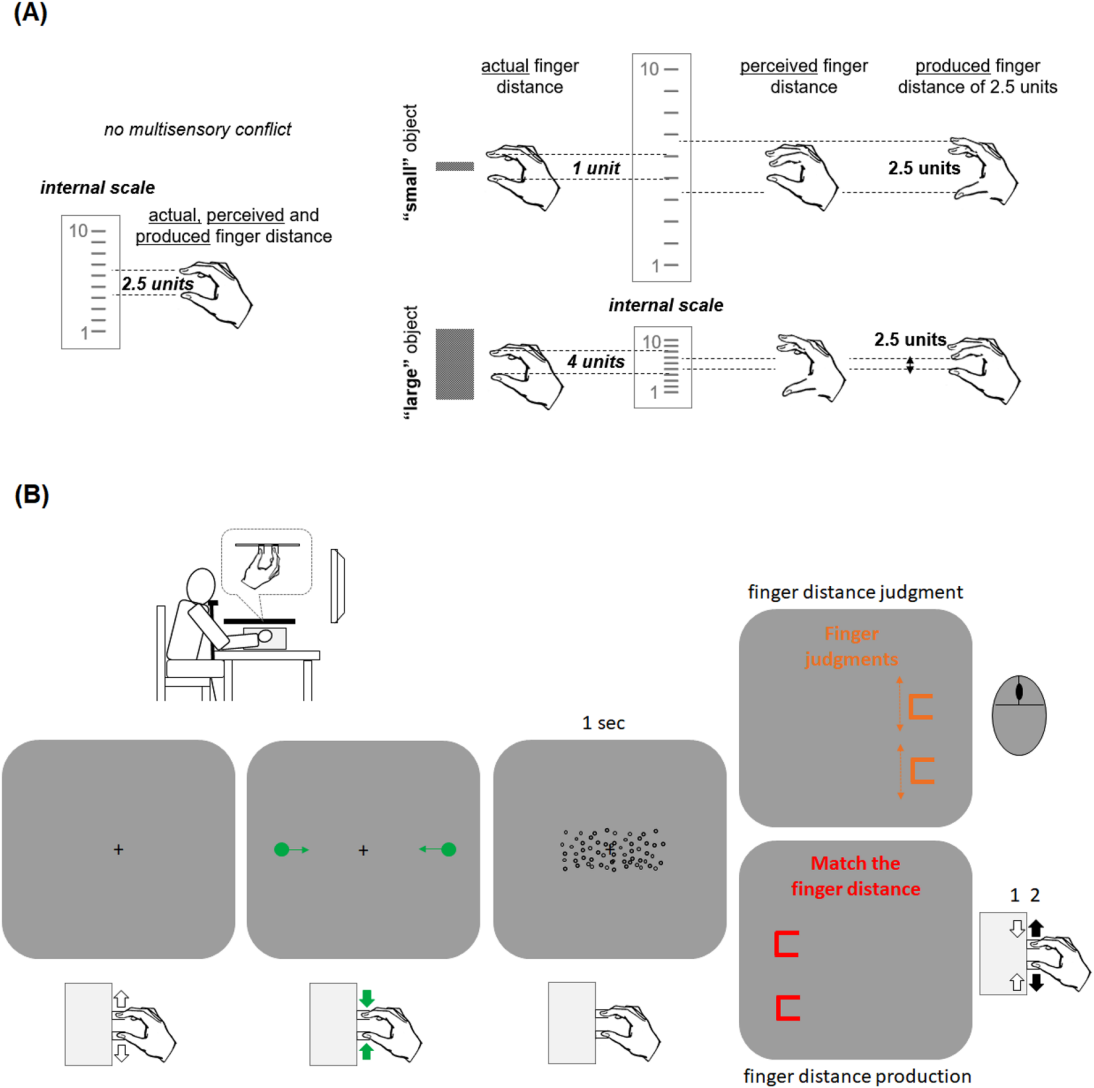
(**A**) A possible relation between changes in body perception indicating multisensory integration (“perceived finger distance”) and those indicating recalibration (“produced finger distance”) in a virtual grasping task. It is assumed that an internal scale of finger distances is expanded and compressed after virtual grasping of smaller and larger objects respectively. This recalibration of the internal reference scale can lead to a decrease (for small objects) and an increase (for large objects) of the perceived finger distance and simultaneously, to larger (for small objects) and smaller magnitudes (for large objects) of a certain produced finger distance. (**B**) Experimental setup and main trial events in Experiments 1. Arrows denote movement directions of visual stimuli and fingers. See main text for further details.

We used a task in which participants virtually enclose visual objects shown in the fronto-parallel plane by a pair of visual cursors controlled by finger movements (see Fig. 1B). In this “virtual grasping task”, the felt finger distance is usually biased towards the current size of the enclosed visual object and, vice versa, the seen size of the object is biased towards the currently felt finger distance (though to a lesser extent). These biases indicate multisensory integration and can be measured by judgments of the adopted finger distance or the size of the object following virtual grasping, e.g.^34,35^. In the present study, participants were asked to align a pair of visual stimuli to a felt finger distance after the target object was virtually enclosed (“finger distance judgment”). This task entails a translation of interoceptive spatial information into visual-spatial information. Yet, to also measure recalibration we asked the participants to align their felt finger distance to a certain visual distance (“finger distance production”; see e.g.^36^ for a similar procedure). This task entails a translation of visual-spatial information into interoceptive spatial information.

We systematically varied the size of the target object and assumed that for a given finger distance an increase in the size of the enclosed target object should be associated with an *increase* of the finger distance judgment, as in our previous studies. This would indicate multisensory integration that creates attraction of spatial information in one sensory modality to judgments of spatial information of another sensory modality. Crucially, according to recalibration, an increase in the size of the enclosed target object should simultaneously lead to a *decrease* of the produced finger distance. Figure 1A delineates why this could be so. Assume that an internal scale of finger distances is somewhat compressed following virtual grasping of large objects and somewhat expanded following grasping of small objects. This could explain why the finger distance feels larger in the former than in the latter condition. In particular, the adopted finger distance now corresponds to more and less units respectively as compared with the original state of the scale prior to an experience of multisensory conflict. If the “labels” (i.e. subjective meaning) of such a scale remain constant, more units would mean a larger, and less units, a smaller finger distance in perception. Simultaneously, when asked to produce a finger distance of a certain magnitude (e.g. 2.5 units), participants should adopt a larger finger distance after grasping a smaller object, and a smaller finger distance after grasping a larger object to compensate for the scale distortions. Consider in analogy two currencies, let say dollar and euro, which typically come with a 1:1 exchange rate. For some reason euros deteriorate, leading to an exchange rate of 1:1.10. From the perspective of euro countries a given good that had cost 100 units in each currency now gets more expensive. Something that had cost 100 euros before, now costs 110 euros. Yet, from the perspective of the United States the same good is getting cheaper. For something that had cost 100 dollars before, now only 91 dollars are needed.

### Methods

#### Participants

Seventeen right-handed participants completed Experiment 1 (12 females and 5 males; *M*_age_ = 24 years, *SD* = 4). They were recruited through the participant pool of the university (SONA systems). Informed consent was obtained from all subjects and/or their legal guardian(s). Each participant received monetary compensation (8 Euro) for her/his participation.

This sample size ensured a power of 0.80 (*α* = 0.05) for effect sizes of about *d* = 0.65 and appeared to be appropriate as our previous similar research demonstrated quite robust effects of visual object characteristics on judgments of hand posture (e.g.^34^; this study revealed an average effect size about d = 0.71).

The study has been approved by the local ethics committee (Ethikkommission des Institutes für Psychologie der Humanwissenschaftlichen Fakultät der Julius-Maximilians-Universität Würzburg, GZEK 2020-67). All methods were performed in accordance with the relevant guidelines and regulations.

#### Apparatus

The experiment was performed in a dark experimental room. Participants were seated in front of a 17′ monitor (NEC MultiSync 1770NX; 1280 × 1024 pixels; 1 pixel = 0.264 mm). The eye monitor distance was about 64 cm. The head was supported by a chin rest. Participants manipulated a finger movement device with their right hand (see the left upper part of Fig. 1B). They had to place their fingers on two metal U-shaped plates that were mirror symmetrically interlocked so that that moving one plate resulted in a mirror-symmetric movement of the other plate. The index finger was placed on one plate, the thumb on the other. To prevent possible exploratory movements of the middle finger during the judgments of finger distance and make the manipulation of the device more comfortable for the participants we also bound the index and the middle fingers of the right hand together. The device had a high spatial resolution (below 0.5 mm), but produced a small constant measurement error (slight increase in overestimation with an increase in distance) that can be assumed to not affect the results substantially. A black cover prevented the vision of the hand and of the movement device. Participants pressed buttons of a computer mouse with the left hand in some trials (see below) to indicate their perceptual decisions. Auditory stimuli were presented through loudspeakers.

#### Stimuli and trial procedure

The main trial events are outlined in Fig. 1B. All stimuli were presented on a gray background (RGB: 128, 128, 128). Each trial started with a small black fixation cross (3 × 3 mm) that indicated that the fingers have to be moved apart from each other. After the distance between the plates (i.e. between the inner sides of the plates) exceeded 7.5 cm a short beep tone was presented and two green cursors appeared (about 2 mm in diameter). Thereafter, the cursor moved along the horizontal in synchrony with but at a different rate as the movements of the fingers. Moving the fingers towards each other caused the cursors to move towards each other and vice versa, moving the fingers away from each other caused the cursors to move away from each other. It is known that visuo-motor mappings can be more or less intuitive and thus be more or less error-prone regarding motor performance^37^. In a previous study, in which a similar virtual grasping task was used and the perception of finger distances was measured, we observed very similar results irrespective of whether the cursors moved along the horizontal or vertical dimension (although a slightly larger bias was observed for the vertical cursor orientation)^34^. Thus, a potential variation in difficulty of the motor task did not seem to play a major role for the used perceptual task and we thus rather arbitrary decided to use the horizontal dimension for the cursors’ movements in the present study.

After a short beep tone was presented and the cursors appeared, the task was to move the fingers together and to place the cursors at the opposed edges of a rectangular target object (i.e. to virtually grab an object by visual movement cursors). When the distance of the cursors to the edges of the target object was less than 5 mm the circles disappeared. The rectangular target object was composed of a number of black unfilled randomly distributed dots (1 mm in size; about 8 dots per cm^2^; see also “Design”) and appeared only when the cursors further approached its edges (i.e. when the centers of the now invisible cursors were less than 2 mm apart from the object’s edges). This virtual grabbing phase was also indicated by a clicking sound that lasted about 1 s if the cursors did not deviate from the edges by 2 mm or more. Participants were asked to maintain this finger distance and to perform corrective movements when the clicking sound disappeared (i.e. when the cursors left the edges of the target object).

Following this object manipulation, participants were asked to estimate the current distance between the fingers in one half of the trials (“***finger distance judgment***”). In these trials, a short text was presented in the upper part of the screen (in orange) indicating that judgments of the current finger distance are required. Moreover, two orange U-shaped objects appeared on the right side (6 × 10 mm [vertical × horizontal] extent; about 8 cm from the center) and the participants had to adjust the distance between these object to the current distance between the fingers. This estimate was done by pressing keys of a computer mouse with the left hand (left/right key produced an increase/decrease of the distance). The judgment was completed by pressing the middle mouse button (scrollwheel). The initial distance between the visual U-shaped objects randomly varied between 50% or 150% of the actual finger distance adopted during object grabbing (i.e. of the current distance between the inner plates of the finger device). If the fingers’ posture of the right hand was changed during the judgments, or if the judgment was confirmed before the initial position of the U-shaped objects was changed, or if the left or the right mouse key was pressed before the judgment phase, then error feedback was presented, and the trial was repeated.

In the remaining trials, participants were asked to *produce* a certain finger distance rather than to judge the current distance adopted during virtual object manipulation (“***finger distance production***”). In these trials, a short text was presented in the upper part of the screen (in red) indicating that the fingers have to be repositioned. Here, participants had to move the fingers together (until a contact of the inner plates) and then to adjust their finger distance to the visual distance between two red U-shaped objects presented on the left side of the screen. These objects were of the same size and had the same offset as the orange objects (see also Fig. 1B). This judgment was also confirmed by pressing the middle mouse button.

#### Design

We slightly varied the finger distance to be adopted during virtual object grabbing between 4.0, 4.2, 4.4, and 4.6 cm. The crucial experimental variable was related to the width of the rectangular target that was either 30% larger or 30% smaller than the finger distance to be adopted (i.e. overall, there were eight widths amounting to about 2.8, 2.94, 3.08, 3.22, 5.2, 5.46, 5.72, and 5.98 cm). Its height was always about 25 mm. In trials including finger distance production, we varied the visual distance between the red U-shaped objects between 2.7, 3.7, 4.9 and 5.9 cm (relates to the distance between the next edges).

The main experiment included 4 blocks with 64 trials each. In each block, 32 trials included a finger distance judgment (4 finger distances to be adopted × 2 rectangle widths × 4 repetitions) and the remaining 32 trials included finger distance production (4 finger distances to be adopted × 2 rectangle widths × 4 to be produced visual distances). The order of all conditions was random. Before the main experiment started, participants performed 10 practice trials that were not analyzed.

#### Analyses and predictions

The data were analyzed using two analyses of variance (ANOVAs) that were performed separately for finger distance judgments and for finger distance production. The main predictions were an increase of finger distance judgments and a decrease of produced finger distances with an increase in the width of the virtually grabbed rectangle. Thus, significant main effects for the factor “width of target object” were expected in both ANOVAs. We also used regression analyses (described in more detail in the results section) to substantiate the main results.

### Results

#### Finger distance judgment

**Figure 2:**
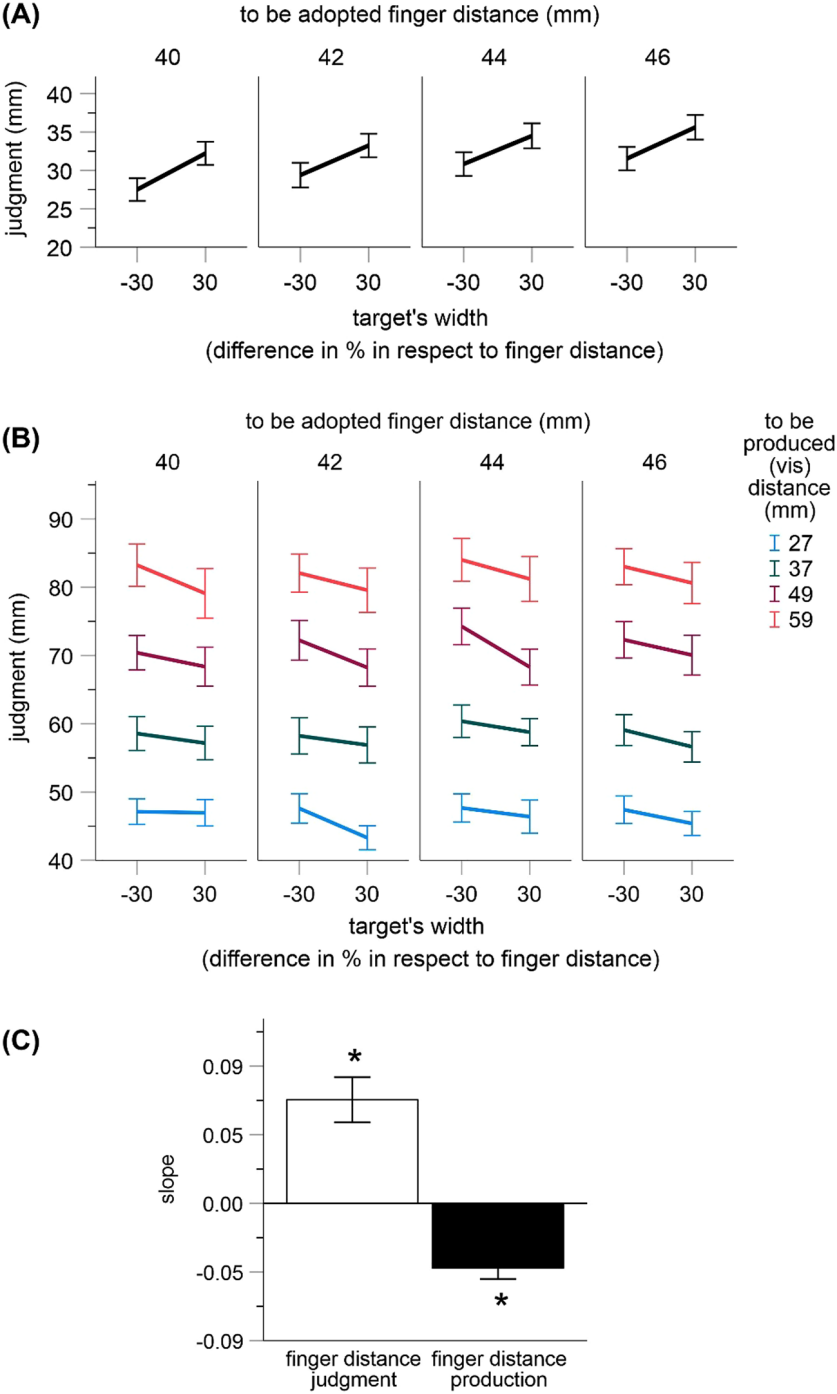
Results of Exp. 1. Shown are mean estimates for all conditions in trials including finger distance judgments (**A**) and finger distance production (**B**) as well as mean coefficients from the regression of finger distance judgments and production (in mm) on the width of targets (in %) (**C**). Error bars are standard errors. Asterisks denote statistical significance (p < 0.05).

As shown in Fig. 2A, virtual grabbing of a larger target object was associated with larger estimates of the finger distance than virtual grabbing of a smaller object. This effect was significant as indicated by a significant main effect for the width of target object in an ANOVA including finger distance to be adopted and the width of target object as within-subjects factors, *F*(1, 16) = 21.26, *p* < 0.001, *η*_*p*_^2^ = 0.571. This is a predicted impact of vision on body-related judgments that we repeatedly observed with this paradigm and that indicates multisensory integration.

The mentioned ANOVA also revealed a significant main effect for the finger distance to be adopted, *F*(3, 48) = 56.46, *p* < 0.001, *η*_*p*_^2^ = 0.779 (other *p* = 0.511). As can be seen in Fig. 2A, the adopted finger distances were generally underestimated and the judgments increased with an increase of the adopted finger distance. The general underestimation bias also occurred in our earlier studies and it resembles systematic errors in body perception that often arise when vision is occluded, e.g.^33,38^. According to Smeets and colleagues^28^ such errors are caused by a change in sensory weighting of visual and proprioceptive information (see also “Introduction”).

#### Finger distance production

As shown in Fig. 2B, virtual grabbing of a larger target object was associated with smaller produced finger distances than virtual grabbing of a smaller object. This predicted effect was similarly pronounced for all visual and adopted finger distances and was expressed in a significant main effect of the width of target object in an ANOVA including finger distance to be adopted, to be produced visual distance and the width of target object as within-subjects factors, *F*(1, 16) = 34.16, *p* < 0.001, *η*_*p*_^2^ = 0.681.

This ANOVA also revealed significant main effects for the finger distance to be adopted and to be produced visual distance, *F*(3, 48) = 4.91, *p* = 0.005, *η*_*p*_^2^ = 0.235, and *F*(3, 48) = 303.66, *p* < 0.001; *η*_*p*_^2^ = 0.950 (other *ps* > 0.140). The produced distances tended to increase with an increase of the adopted finger distance. Also, the produced distances varied as a function of the visual distances (Fig. 2B). Notably, participants substantially overestimated visual distances. We assume that this bias arose due to an adjustment of motor behavior to the systematic underestimation of felt finger distances mentioned above (i.e. if a given finger distance feels smaller one has to move the fingers further apart to produce a certain visual distance).

#### Regression analysis

To substantiate these results we also performed two regression analyses. The width of target object (in %) served as a predictor (i.e. − 30% or + 30%). The judged finger distances (finger distance judgments) or the produced finger distances (finger distance production) served as dependent measures (averaged over the adopted and produced distances for each participant). The (unstandardized) regression coefficients indicating the slopes of the regression lines were positive for finger distance judgments (Mean = 0.068, 95% CI [0.037, 0.099]), and negative for finger distance production (Mean = − 0.042, 95% CI [− 0.058, − 0.027]; see Fig. 2C). Both were significantly different from zero, *t*(16) = 4.61, *p* < 0.001 and *t*(16) = 5.84, *p* < 0.001 (two-tailed, adjusted α = 0.025). This result suggests that finger distance judgments systematically increased, whereas the produced finger distances systematically decreased with an increase in target width as predicted.

## Experiment 2

Exp. 2 aimed to conceptually replicate the results of Exp. 1 under changed stimulus conditions. It included more levels of multisensory discrepancies and used another task to measure recalibration. In particular, we here used four different sizes for the to be grabbed target object for each of two adopted distances. Moreover, we now asked the participants to align their finger distance to certain cm values after virtual grasping to measure recalibration. The main difference to Exp. 1 here was thus the absence of visual extent on the screen during the judgment procedure (i.e. of the U-shaped objects shown during the judgment). This change allowed us to evaluate whether the varying size of the visual target affected the perception of the visual extent on the screen, rather than of the felt finger distance (see also Exp. 3). The general rationale and hypotheses were the same as in Exp. 1.

### Methods

#### Participants

Twenty-one right-handed participants were recruited for Exp. 2 (18 females and 3 males; *M*_age_ = 26 years, *SD* = 5). Informed consent was obtained from all subjects and/or their legal guardian(s). Participants received monetary compensation (10 Euro) for their participation.

The study has been approved by the local ethics committee (Ethikkommission des Institutes für Psychologie der Humanwissenschaftlichen Fakultät der Julius-Maximilians-Universität Würzburg, GZEK 2020-67). All methods were performed in accordance with the relevant guidelines and regulations.

#### Apparatus

The apparatus was the same as in Exp. 1 except for a small technical issue that emerged at a certain point during the data collection. In particular, due to mechanical reasons the finger movement device constantly provided about 2 mm smaller values of finger distances (the scale of values was marginally displaced after data collection). That is, the data of some participants include such a systematic underestimation bias.

#### Stimuli and trial procedure

Before each block of trials the screen was filled with randomly distributed black filled dots (1 mm in size; 4 dots per cm^2^). The initial virtual grabbing phase of the trial procedure was the same as in Exp. 1. That is, participants moved their fingers apart and then together to encompass a rectangular target object by a pair of visual cursors (see the first three displays in Fig. 1B). Also, participants were asked to estimate their current finger distance following virtual object grabbing in one half of the trials (“***finger distance judgment***”). In these trials, the German word for “fingers” was presented in the upper part of the screen (in orange) and two orange U-shaped objects appeared on the right side. The participants adjusted the distance between these object to the current finger distance pressing keys of a computer mouse as in Exp. 1 (see Fig. 1B, right upper part). In the remaining trials, participants were asked to produce a certain finger distance (“***finger distance production***”). In these trials, a short text including a distance in cm was presented in the upper part of the screen (in red). Participants had to move the fingers together and then to adjust their finger distance to the distance indicated by the text. The rest of the trial procedure was as in Exp. 1.

#### Design

In Exp. 2, there were two finger distances to be adopted during virtual object grabbing, 3.7 and 4.3 cm. These distances corresponded to the height of the target object of 4.3 and 3.7 cm respectively. The width of the target object could be 15 and 45% larger or smaller than the finger distance to be adopted (i.e. for each finger distance and object’s height there were 4 widths of the target object). In trials including finger distance production, the to be produced finger distance varied between 1, 3, 5 and 7 cm.

The experiment included 4 blocks with 64 trials each. In each block, 32 trials included a finger distance judgment (2 finger distances to be adopted × 4 rectangle widths × 4 repetitions) and the remaining 32 trials included finger distance production (2 finger distances to be adopted × 4 rectangle widths × 4 to be produced finger distances). The order of all conditions was random. Before the main experiment started, participants performed 10 practice trials that were not analyzed.

#### Analyses and predictions

The analyses and predictions were as in Exp. 1. That is, two ANOVAs were performed separately for finger distance judgments and for finger distance production and significant main effects for the factor “width of target object” in both ANOVAs were expected. Regression analyses were again used to substantiate the main results.

### Results

#### Finger distance judgments

**Figure 3:**
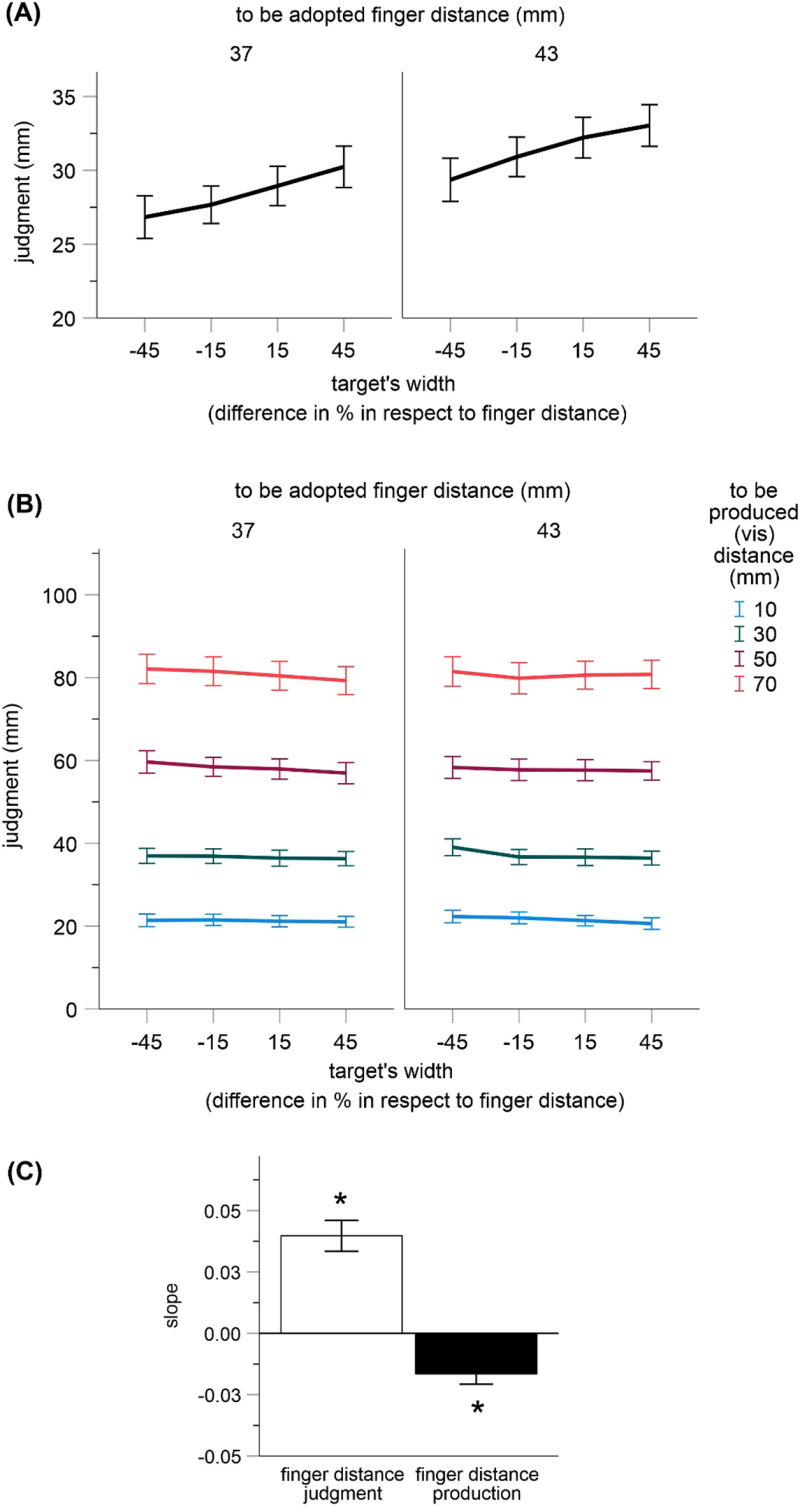
Results of Exp. 2. Shown are mean estimates for all conditions in trials including finger distance judgments (**A**) and finger distance production (**B**) as well as mean coefficients from the regression of finger distance judgments and production (in mm) on the width of targets (in %) (**C**). Error bars are standard errors. Asterisks denote statistical significance (p < 0.05).

As shown in Fig. 3A, the wider the target object was, the larger was the judgment of the finger distance to be adopted. This effect was significant as indicated by a significant main effect for the width of target object in an ANOVA including finger distance to be adopted and the width of target object as within-subjects factors, *F*(3, 60) = 23.40, *p* < 0.001, *η*_*p*_^2^ = 0.539. This is again a replication of the impact of vision on body-related judgments indicating multisensory integration.

The ANOVA also revealed a significant main effect for finger distance to be adopted, *F*(1, 20) = 117.75, *p* < 0.001, *η*_*p*_^2^ = 0.855 (other *p* = 0.473), indicating that the judgments increased with an increase of the adopted finger distance.

#### Finger distance production

The judgments systematically decreased with an increase in width of the virtually grabbed visual object. This predicted effect was similarly pronounced for all to be produced and adopted finger distances and was expressed in a significant main effect of the width of target object in an ANOVA including finger distance to be adopted, to be produced distance and the width of target object as factors, *F*(3, 60) = 5.45, *p* = 0.002, *η*_*p*_^2^ = 0.214. This outcome is a replication of the results of Exp. 1 in the distance production trials. It indicates an impact of virtual grasping on body-related recalibration and suggests that the results of Exp. 1 are not due to an impact of target size on the perception of visual extent on the screen.

The ANOVA also revealed a significant main effect for the factor to be produced distance, *F*(3, 60) = 297.36, *p* < 0.001; *η*_*p*_^2^ = 0.937 (no other effects were significant, *ps* > 0.427). The produced distances varied as a function of the to be produced distances (see Fig. 3B; see also Exp. 1).

#### Regression analysis

We again performed two regression analyses. The width of target object served as a predictor (in %) and the judged finger distances (finger distance judgments) or the produced finger distances (finger distance production) as dependent measures (averaged over the adopted and produced distances for each participant). The resulting regression coefficients were positive for finger distance judgments (Mean = 0.040, 95% CI [0.027, 0.053]), and negative for finger distance production ((Mean = − 0.017, 95% CI [− 0.025, − 0.008]); see Fig. 3C). Both were significantly different from zero, *t*(20) = 6.30, *p* < 0.001 and *t*(20) = 3.91, *p* = 0.002 (two-tailed, adjusted α = 0.025), suggesting that finger distance judgments systematically increased, whereas the produced finger distances systematically decreased with an increase in target width as predicted.

## Experiment 3

With Exp. 2, we could replicate the results of Exp. 1 under changed stimulus conditions suggesting that the main pattern of results is reliable. One could question, however, whether the method we used measured body perception. In particular, the varying size of the visual target could have influenced the judgement of the visual distance on the screen (i.e. of the distance between the visual U-shaped objects shown during the judgment), rather than of the felt finger distance. For the recalibration task (i.e. for finger production), this possible concern can be ruled out, as the results of Exp. 1, in which U-shaped objects were used, were replicated in Exp. 2, in which no U-shaped objects were used and the participants adjusted their finger posture to a certain cm value shown on the screen. For the multisensory perception task (i.e. for finger distance judgment), in contrast, a purely visual influence could, in theory, explain the observed results. This is, however, very unlikely as we previously consistently observed a perceptual attraction of the felt finger distance by visual target size in virtual grasping under diverse conditions including those where a purely visual effect could be ruled out, e.g.^34^, see also^10–14^ for related results. Nevertheless, with Exp. 3 we aimed to demonstrate that the size of the visual target affects the judgments of finger distance as in Exp. 1 and 2 also when a purely visual effect can be ruled out.

For this purpose, we repeated the finger distance judgement condition of Exp. 1 but did not use the visual (U-shaped) markers during the judgment procedure. Instead, we asked the participants to judge their felt finger distance in mm. If the results of Exp. 1 and 2 can be replicated in Exp. 3, then the method used in Exp. 1 and 2 can be considered as a valid measure of body perception under the present conditions.

### Methods

#### Participants

Twelve right-handed participants were recruited for Exp. 3 (10 females and 2 males; *M*_age_ = 26 years, *SD* = 5). Informed consent was obtained from all subjects and/or their legal guardian(s). Participants received monetary compensation (6 Euro) for their participation.

This sample size ensured a power of 0.80 (*α* = 0.05) for effect sizes of about *dz* = 0.8 and appeared to be appropriate as the results of Exp. 1 and 2 demonstrated quite robust effects (e.g. Exp. 1 revealed an effect size of *dz* = 1.33 that would require 6 participants given an *α* of 0.05 and a power of 0.80).

The study has been approved by the local ethics committee (Ethikkommission des Institutes für Psychologie der Humanwissenschaftlichen Fakultät der Julius-Maximilians-Universität Würzburg, GZEK 2020-67). All methods were performed in accordance with the relevant guidelines and regulations.

#### Apparatus

The apparatus was the same as in Exp. 1 and Exp. 2 except that keyboard illuminated by a table lamp was used to collect perceptual judgments. Due to mechanical reasons the scale of values of the finger movement device was slightly displaced after data collection (i.e. the data of some participants included a small constant error; see also Exp. 2).

#### Stimuli and trial procedure

Stimuli and trial procedure were the same as in Exp. 1 except for the following changes. First, the finger distance production trials were omitted. Second, in the finger distance judgments trials participants were asked to estimate the current distance between the fingers in mm. In these trials, a short textual prompt (“Estimate the finger distance in mm” in German) was presented slightly above the middle of the screen (in orange) after the virtual grabbing phase. In response to this stimulus, participants had to type the corresponding number using a keyboard and to confirm their estimate by pressing the enter key (corrections were possible using the backspace key).

#### Design

Except for the finger production trials that were omitted in Exp. 3 the design was the same as in Exp. 1. That is, there were four finger distances to be adopted (4.0, 4.2, 4.4, and 4.6 cm) and eight widths of the target object (30% larger and 30% smaller than each adopted finger distance). The main experiment included 4 blocks with 32 trials each. The order of all conditions was random. Before the main experiment started, participants performed 10 practice trials that were not analyzed.

#### Analyses and predictions

Trials with no judgments and with judgments above 10 cm (i.e. trials with alleged typing errors) were excluded from analysis (0.26% of trials). The mean data were analyzed using an ANOVA with adopted finger distance and the width of target object as factors. The main prediction was an increase of finger distance judgments with an increase in the width of the target. Thus, a significant main effect for the factor “width of target object” were expected in the ANOVA.

### Results

**Figure 4:**
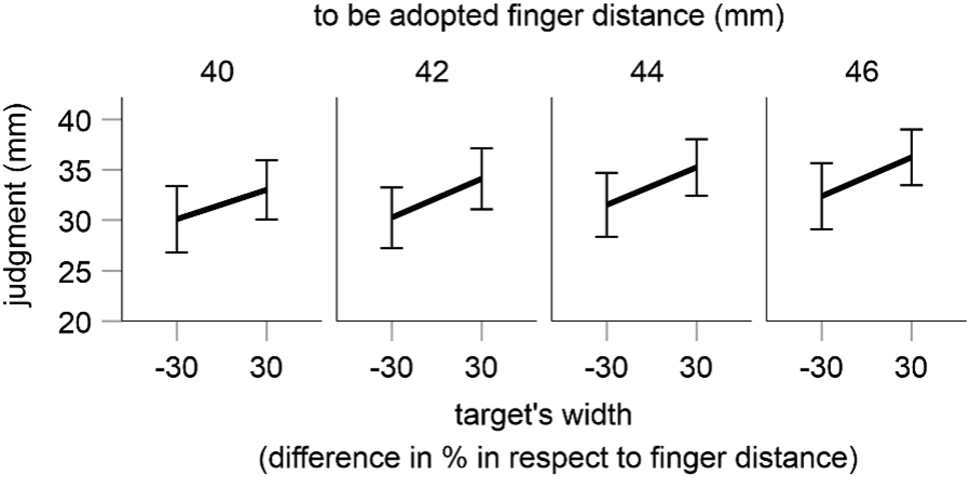
Results of Exp. 4. Shown are mean estimates for all conditions. Error bars are standard errors.

As shown in Fig. 4, finger distances were judged as larger after virtual grabbing of a larger target object than after grabbing of a smaller object. This effect was significant as indicated by a significant main effect of the factor width of target object in an ANOVA including finger distance to be adopted and the width of target object as within-subjects factors, *F*(1, 11) = 8.44, *p* = 0.014, *η*_*p*_^2^ = 0.434. This result is a replication of the main results observed in the finger judgment trials of Exp. 1 and 2. It indicates an impact of vision on body-related perception in the virtual grasping task and suggests that the results of Exp. 1 and 2 are not due to some purely visual factors.

The mentioned ANOVA also revealed a significant main effect for the finger distance to be adopted, *F*(3, 33) = 12.90, *p* < 0.001, *η*_*p*_^2^ = 0.540 (other *p* = 0.559). As can be seen in Fig. 4, and in accordance with Exp. 1 and 2, the adopted finger distances were generally underestimated and the judgments increased with an increase of the adopted finger distance.

## Discussion

The present study examined whether changes in body perception usually ascribed to multisensory integration processes are accompanied by changes in body perception that are usually ascribed to processes of sensory or motor recalibration. We observed that after virtual grasping of larger objects a given finger distance was perceived as larger than after grasping of smaller objects. This repeatedly observed result indicates multisensory integration, e.g.^34^. Simultaneously, after virtual grasping of a larger object a given to be produced finger distance was underestimated as compared to after virtual grasping of a smaller object. This novel observation indicates that an interoceptive scale was altered or recalibrated in a way that can also explain the multisensory biases. Thus, changes in perception indicating multisensory integration and those indicating recalibration could have the same origin under the present conditions as we suggest in Fig. 1A.

This conclusion should of course be considered with caution. In particular, the effects observed for finger judgments were substantially smaller than those observed for finger production. This could speak for the involvement of different mechanisms. Note, however, that the methods used to measure multisensory perception and recalibration are usually not the same due to historical reasons: perception is measured rather immediately following a multisensory conflict, whereas recalibration is measured not until an additional unisensory stimulus is presented (consider e.g. the additional finger movements in our procedure). This methodical difference could entail a decay of the crucial conflict information and could thus potentially account for the smaller magnitude of the observed effects in the recalibration condition.

A close relation between perceptual biases following sensory integration and sensorimotor learning has also been often proposed in earlier studies^9,39,40^. However, more recent research on this issue did not reveal a consistent picture. For example, the reliability weighting rule has been assumed to hold for recalibration by some authors^41–43^. Other studies, in contrast, argued that recalibration does not depend on sensory reliabilities and indicated that perceptual accuracy rather than reliability is important for recalibration^44–46^. In a similar vein, there is evidence indicating a shared neurophysiological mechanism between perception and learning dependent biases^24^. Other studies rather speak for their independence^23,25,26^. Thus, the issue is not well understood yet and the results seem be depend on task and context conditions^47^. Against this background, the observed coincidence of biases indicating integration and recalibration could be not generally valid.

It should also be noted that we used a motor task to measure recalibration in that participants actively aligned their fingers with some distances, see also^36^. We can thus not dissociate whether the effects observed in the recalibration condition reflect changes in a sensory (i.e. proprioceptive) or in a motor (i.e. motor command) map, or in both, e.g.^48^. In a first approximation, we assumed that changes in perception and motor behavior are interrelated and arise from the same source (i.e. from changes in a common reference scale). This assumption is in line with some previous suggestions^16^ but contradicts some others^49^. Thus, the main conclusion regarding the inferred changes in the common internal scale is valid to the extent this assumption is empirically supported.

To sum up, the present results indicate that perceptual biases that arise following multisensory integration and those that occur in the course of sensorimotor learning during interactions with the environment rely on similar mechanisms.

## Data Availability

The data have been made publicly available via the Open Science Framework and can be accessed at https://osf.io/7s4za/.
